# Hydrogen and Methane Breath Test in the Diagnosis of Lactose Intolerance

**DOI:** 10.3390/nu13093261

**Published:** 2021-09-18

**Authors:** Charlotte De Geyter, Kris Van de Maele, Bruno Hauser, Yvan Vandenplas

**Affiliations:** KidZ Health Castle, Vrije Universiteit Brussel (VUB) UZ Brussel, 1090 Brussels, Belgium; Charlotte.DeGeyter@uzbrussel.be (C.D.G.); Kristel.VanDeMaele@uzbrussel.be (K.V.d.M.); bruno.hauser@uzbrussel.be (B.H.)

**Keywords:** lactose intolerance, hydrogen breath test, methane

## Abstract

The hydrogen (H_2_) breath test is a non-invasive investigation used to diagnose lactose intolerance (LI). Patients with LI may also expire increased amounts of methane (CH_4_) during a lactose test. The aim of this study is to evaluate the contribution of CH_4_ measurements. We tested 209 children (1–17 years old) with symptoms suggesting LI with lactose H_2_ and CH_4_ breath tests. The result was positive when the H_2_ excretion exceeded 20 parts per million (ppm) and the CH_4_ was 10 ppm above the baseline. A clinician, blinded for the results of the breath test, registered the symptoms. Of the patient population, 101/209 (48%) were negative for both H_2_ and CH_4_; 96/209 (46%) had a positive H_2_ breath test result; 31/96 (32%) were also positive for CH_4_; 12/209 (6%) patients were only positive for CH_4_. The majority of hydrogen producers showed symptoms, whereas this was only the case in half of the H_2_-negative CH_4_ producers. Almost all patients treated with a lactose-poor diet reported significant symptom improvement. These results indicate that CH_4_ measurements may possibly be of additional value for the diagnosis of LI, since 5.7% of patients were negative for H_2_ and positive for CH_4_, and half of them experienced symptoms during the test.

## 1. Introduction

Lactose intolerance is defined as the occurrence of symptoms such as abdominal pain, bloating, and diarrhea after the ingestion of lactose in patients with lactose malabsorption. Although congenital and secondary lactase deficiencies occur, most children suffer from a “primary late onset” [1] or “adult type” lactase deficiency due to the non-persistence of the enzyme lactase-phlorizin hydrolase after childhood [2].

Adequate amounts of lactase, present in the villi of the small intestinal brush border, are required for the digestion of lactose. However, if the lactase activity is insufficient, undigested lactose is fermented in the colon by anaerobic bacteria. In that case, lactose is metabolized into carbon dioxide (leading to bloating), methane (CH_4_), propionic and butyric acids (short-chain fatty acids, leading to osmotic diarrhea), and hydrogen (H_2_). Gasses such as H_2_ and CH_4_ accumulate in the intestine, but also pass through the intestinal wall into the bloodstream and are transported to the lungs, where they are excreted in the exhaled breath [3]. In humans, the only source of H_2_ and CH_4_ is their production by bacterial metabolism of carbohydrates [4].

When anamnesis is suggestive for lactose intolerance, a H_2_ breath test is considered the golden standard for the diagnosis, as it is a safe, cost-effective, and non-invasive investigative tool [5] with a high sensitivity (70–100%) and specificity (69–100%) [6]. A meta-analysis by Marton, Xue, and Szilagyi [7] comparing the diagnostic accuracy of lactose H_2_ breath tests found an overall sensitivity of 0.88 (CI 0.85–0.90) and specificity of 0.85 (CI 0.82–0.87). A H_2_ increase of 20 ppm above the baseline level is considered a positive test result, as suggested by the Rome Consensus Conference. In pediatric populations, a test duration of 3 h is advised, with samples taken at intervals of at least 30 min [8].

However, 2.5–15% of the H_2_ breath test results are false-negative due to a variety of causes, such as non-producers of H_2_ or increased production of CH_4_ [9,10,11]. It is known that the production of one mole of CH_4_ consumes four moles of H_2_ and one mole of carbon dioxide, thereby also reducing the intracolonic gas content [12]. From this perspective, multiple studies examined the concomitant determination of CH_4_ next to H_2_ in order to improve the diagnostic accuracy of the H_2_ breath test. A study by Vernia et al. [13] reported higher rates of false negative results in patients with predominant CH_4_ production, with a CH_4_ cut-off value of 10 ppm above the baseline, compared to patients with low CH_4_ production. In other studies, significantly increased breath CH_4_ levels were found in both lactose intolerant children [14] and adults [15], and the combined breath test for H_2_ and CH_4_ is considered more appropriate for the diagnosis of lactose malabsorption [16]. However, other authors did not find any advantage of the concomitant measuring of CH_4_ [17]. Due to a lack of conclusive results in literature, the routine measurement of breath CH_4_ excretion is not currently advised, although it is suggested in the Rome Consensus that measuring CH_4_ excretion might improve diagnostic accuracy [8].

The aim of this pilot study is to evaluate the importance of the combined measurement of H_2_ and CH_4_ in the diagnosis of lactose intolerance.

## 2. Materials and Methods

Data were collected from November 2019 to July 2020 in the pediatric gastroenterology daycare unit of the KidZ Health Castle. Subjects were patients <18 years old, referred by a pediatrician for a lactose breath test because of reported symptoms suggestive of lactose intolerance. Parents and/or children (when old enough) gave permission to use the test results for this analysis. The history of the included patients was checked for the absence of evidence of small intestinal bacterial overgrowth, celiac disease, non-celiac gluten hypersensitivity, food allergy, or gastrointestinal infectious disease. Celiac disease was excluded because of the normal transglutaminase IgA antibodies in IgA sufficient children. The anti-gliadin IgG was within normal ranges in all children. The specific IgE for the common food allergies (egg white, cow milk, soy, fish, wheat, and gluten) was within normal ranges. The stool cultures, including parasites, were negative.

The tests were performed in the pediatric gastroenterology day-clinic after an overnight fasting time of at least 6 h. A basal H_2_ measurement below 10 ppm was set as a control of the fasting state. The pre-test conditions, according to the 2009 Rome Consensus Conference [8], were respected. Exercise and smoking before and during the test were avoided. The exclusion criteria were children who were not presumed healthy, or had a history of probiotic or antibiotic intake during the two weeks preceding the H_2_ breath test [18].

The lactose was administered orally (a dose of 2 g/kg, maximum 50 g) and diluted in a maximum of 250 mL of water. The expired air was collected in specific gas-tight syringes with a capacity of 60 mL. One breath sample was taken before the intake of lactose, and subsequent breath samples were collected in a sitting position every 15 min in a 3 h period after the ingestion of lactose. The patients were asked to remain in the waiting room and to avoid physical activity. The expired H_2_ and CH_4_ were measured with a specific analyzer (Microlyzer DP; Quintron Instruments, Milwaukee, WI, USA). The system was calibrated daily.

The result was considered positive when a H_2_ peak exceeded 20 ppm above the baseline. The CH_4_ excretion was considered positive when reaching 10 ppm above the baseline.

Gastro-intestinal symptoms such as abdominal pain, bloating, diarrhea, as well as extra-intestinal symptoms such as fatigue, headache, and dizziness, were registered every 15 min when a breath sample was collected. This was performed by a nurse, blinded for the results of the breath test.

The analyses were performed using SPSS version 26. The statistical significance of the differences between the percentage of participants who were CH_4_ producers in various groups was determined by chi^2^ analysis. The significance of the correlations between the CH_4_ and H_2_ breath concentrations of producers vs. age was determined using the Spearman nonparametric correlation coefficient, because breath concentrations were not distributed normally.

## 3. Results

We tested 209 children with symptoms suggesting lactose intolerance with a lactose H_2_ and CH_4_ breath test. The mean age of our patients was 8.3 years (range 1.1–17.3 years, median 8.4 years, SD ± 3.78), including 56% girls and 44% boys. Over 90% reported gastrointestinal complaints, predominantly cramps or abdominal pain, flatulence, bloating, and diarrhea. In addition to the gastrointestinal complaints, a small proportion (~5%) also reported systemic complaints such as headaches.

In this group, 96 children (46%) had a positive H_2_ breath test, of which 58% were girls. Of the female population, 65 of them (67.7%) tested positive for H_2_, but showed no increased production of CH_4_. On the other hand, 31 out of 96 (32.3%) patients had a positive test result for both H_2_ and CH_4_ excretion. In the group of CH4 producers, 48% were girls. It is interesting to note that 28% (12/43) of the CH_4_ producers had a negative result for H_2_ production. Children were classified with malabsorption when the breath test was positive but the child continued to show no symptoms. The rise in H_2_ or CH_4_ within the first hour did not occur in any of the children, which may have been indicative of small intestinal bacterial overgrowth [19].

Significantly more CH_4_ producers were present in the group of H_2_ producers (Table 1; 5.7 vs. 14.8%; CHI square < 0.001), which is in line with the theory that elevated amounts of H_2_ are necessary for production of CH_4_. In 10 children excreting high amounts of CH_4_ (>20 ppm above baseline), 6 of them also tested positive for the H_2_ test. Only 2 of these 10 children were younger than 8 years old, suggesting that age might play a significant role in methanogenesis. Furthermore, we found age to be correlated significantly with baseline CH_4_ levels (Spearman.149, *p* = 0.031) and with maximum CH_4_ (Spearman.142, *p* = 0.040), but not with H_2_ values.

Prior to the administration of lactose, we found a mean breath H_2_ excretion of 19.7 ± 20.9 and a net CH4 excretion of 18.2 ± 11.2 (Table 2). No significant differences were seen in the baseline values of H_2_ or CH_4_ between normal and lactose malabsorbing children. After the administration of lactose, high elevations of breath H_2_ (Delta H_2_ > 100) were seen, as expected in the lactose-intolerant children, but also in CH4 producers.

Significant correlations were found between delta H_2_ and delta CH_4_ values (P.358, *p* < 0.001), delta H_2_ and max CH_4_ values (P.191, *p* = 0.005), and between delta CH_4_ and max H_2_ values (P.312, *p* < 0.001). This suggests that the elevations in H_2_ and elevations in CH_4_ coexist; this supports the hypothesis that CH_4_ production is dependent on a (minimum of) H_2_ production by conversion.

Overall, the difference in timing between CH_4_ and H_2_ peaks did not reach significance (median of 105 H_2_ vs. 120 CH_4_ min, *p* = 0.058). There was no influence of sex (*p* = 0.20) or age (*p* = 1).

In total, 82 patients were treated with a lactose-free diet, and 56 (68.3%) were re-evaluated after 4 to 6 weeks. All but three patients reported significant improvement with the diet. These three patients had CH_4_ starting values between 16 and 34 ppm; one patient also had a H_2_ baseline value >20 ppm. At the follow-up consultation, the lactose-free diet was changed to a lactose-poor diet according to individual tolerance, and further follow-up was done by the referring physicians.

## 4. Discussion

In our study population, 14.8% of the children with a positive H_2_ breath test (>20 ppm above baseline) were also positive for CH_4_ (>10 ppm above baseline). Moreover, we detected significant CH_4_ production in 5.7% of the children with a negative H_2_ test, which is in line with the amount of false negatives (2.5–15%) previously reported [9,10,11]. Houben et al. reported that the additional measurement of CH_4_ (considering an increase with >5 ppm as positive) improved the accuracy of the test, as 16% of subjects (out of 1051 patients, with 178 children) with normal lactose digestion and no H_2_ excretion were found to excrete CH_4_ [20]. In this study, a rise in ^13^CO_2_ excretion was used as a standard to diagnose lactase deficiency [20]. Based on a retrospective analysis of 282 breath tests, Peron et al. concluded that merging H_2_ and CH_4_ stoichiometric values resulted in an increased sensitivity [21].

Almost all (53/56) children in our study indicated fewer gastrointestinal complaints when on a lactose-free diet, suggesting that they likely did suffer from lactose intolerance.

As excessive CH_4_ production is thought to cause more health consequences than an excess production of H_2_ [22], it is of high importance to detect these subjects. The CH_4_ levels in expired air are suggested to be related to constipation in patients with irritable bowel syndrome [22]. This hypothesis is indirectly endorsed by our finding that only half of the CH_4_ producers who did not produce H_2_ showed symptoms suggesting lactose intolerance, whereas this was the case in 80–90% of the H_2_ producers. However, some authors point out the difficulty of using reported symptoms during breath tests in the diagnosis of lactose intolerance, as they are often not reliable and/or poorly correlated [23,24].

Recently, instruments for the simultaneous measurement of CH_4_ and H_2_ have become more widely available for clinical use. Normally, patients produce either H_2_ or CH_4_, and co-producers were rarely identified when using older instruments [25]. However, with more modern detection systems, co-production is detectable even at low levels [22]. Ruzsanyi et al. [23] reported elevated CH_4_ levels in a majority of the children with a positive H_2_ breath test. Furthermore, since it is suggested that excessive CH_4_ production may cause more health consequences than an excess of H_2_ [22], it becomes even more important to measure CH_4_. In our study, 46% of the children demonstrated lactose malabsorption with a positive H_2_ breath test (of which 83% also reported symptoms compatible with LI). These data are comparable to other results in children [14,23], showing that 38% and 36%, respectively, of the tested children had a positive H_2_ breath test. The ratio of boys and girls in the group with a positive H_2_ test was identical to the ratio of the entire study group.

Background atmospheric CH_4_ is about 1.7 ppm. The subjects excreting more than 1 ppm of CH_4_ above the environmental value [26] are considered breath CH_4_-producers. Therefore, a threshold of 3 ppm was proposed [27]. Others proposed a threshold of 10 ppm [19,23], and in a recent study by Hammer et al. [28], malabsorption was defined by a net increase of ≥5 to ≥12 ppm for CH_4_, and ≥10 to ≥15 ppm for H_2_ plus CH_4_. In our group, 25% of the children showed elevated CH_4_ excretion of more than 10 ppm. It was stated that patients usually produce either H_2_ or CH_4_, and only rarely produce both [25]. Hammer et al. [28] also found that the addition of CH_4_ hardly influenced the results of malabsorption, and only under the use of specific cut-offs (the combined rise in H_2_ and CH_4_ should be less than 18) was a significant increase in the rate of malabsorbers seen. However, in our study, most of the CH_4_-positive tests (72%) were found in the children that also demonstrated a positive H_2_ breath test. Medow et al. [14] also reported significantly increased breath CH_4_ levels in lactose intolerant children, although they already considered a H_2_ test positive from 10 ppm above baseline. Schneider et al. [29] found a significant correlation between CH_4_ and H_2_ breath tests (Fisher’s exact test, *p* < 0.001), with a strong relationship (Phi coefficient: φ = 0.84, *p* < 0.001) and excellent agreement (Cohen’s κ = 0.837; 95% CI 0.682–0.992; *p* < 0.001). Furthermore, they stated that it might be possible to detect non-hydrogen producers using a lower CH_4_ cut-off value (5 ppm), as it resulted in 26% more positive CH_4_ breath tests. Ruzsanyi et al. [23] reported elevated CH_4_ levels in a majority (83%) of the children with a positive H_2_ breath test. It should be noted that in these studies, CH_4_ levels were considered to be elevated when exceeding the baseline by only 1 ppm. They reported elevated CH_4_ concentrations (>10 ppm above baseline) in 15% of the population, and an additional 28% of values greater than 1 ppm above the baseline were seen. However, it must be noted that most of them already showed elevated values before the ingestion of lactose, which suggests that other factors are contributing to methanogenesis (e.g., small intestinal bacterial overgrowth (SIBO) [22], constipation [30], or constipation-predominant IBS [31]). Recently, Gottlieb et al. [32] confirmed significantly elevated CH_4_ levels in SIBO (confirmed with a lactulose breath test) and even proved a single-time-point CH_4_ breath sample taken after an overnight fast (without administration of substrate) to be equally accurate in diagnosing SIBO. There is also evidence that CH_4_ producers experience slower transit times [33], and higher CH_4_ excretion levels are seen in subjects suffering from constipation [34]. We found slightly later peak values in CH_4_ (120 min) compared to H_2_ peaks (105 min), but this difference was not significant (*p* = 0.058). Possibly significant elevations of H_2_ could still occur after the test registration (after 180 min).

The CH_4_ producing status depends on many influencing factors, such as age [35], sex [36], ethnic background [37,38], exercise [39], and gastrointestinal diseases [22,38]. CH_4_ is rarely identified in the breath of subjects less than three years of age [40,41,42], which is probably due to a later acquisition of a methanogenic microbiome [14]. CH_4_ production is considered to increase with age in an approximately linear way [35] until the adult distribution is reached [26]. However, others did not find any correlation between age and exhaled maximum CH_4_ concentration in children [23]. Furthermore, primary late onset lactose intolerance is characterized by a gradual reduction in lactase activity, and it generally manifests itself only after the age of 5 to 6 years of age in white populations, although it can occur earlier in predominantly non-white populations [1]. In general, it does not occur before the age of 2 years, which may explain why CH_4_ production and excretion is rarely seen in this age category. We found age to be correlated significantly with the start CH_4_ values (P.149, *p* = 0.031) and with the maximum CH4 values (P.142, *p* = 0.040), but not with H_2_ values. However, some authors reported CH_4_ production already present in infants and toddlers [25,43].

Several authors mention a female dominance in CH_4_ producers, certainly in young women [35,36], but other studies did not find any significant difference between male and female CH_4_ producers [26,44], and little is known about sex differences in CH_4_ excretion in children. No significant differences based on sex could be found in our study.

Ethnic differences were not taken into account in our study, which is a shortcoming, as it might influence the CH_4_-producing status [37,38]. Furthermore, since the lactose-free diet intervention was open, and a blinded lactose challenge was not performed, a placebo effect cannot be excluded.

## 5. Conclusions

Although previous publications concluded that the addition of CH_4_ determination to the standard H_2_ measurement increases the sensitivity of a lactose breath test, the contribution of CH_4_ is still debated and not routinely performed. Our results confirm that the concomitant measurement of CH_4_ is of additional value for the diagnosis of LI, since 5.7% of the children showed only an elevation of expired CH_4,_ and half of these children were symptomatic during the breath test. Further research is needed before recommending systematic CH_4_ measurements.

## Figures and Tables

**Table 1:** Hydrogen (H_2_) versus methane (CH_4_) results.

	H_2_ +	H_2_ −	Total
CH_4_ +	31 (14.8%) M: 3 I: 28	12 (5.7%) M: 6 I: 6	43 (20.6%)
CH_4_ −	65 (31.1%) M: 13 I: 52	101 (48.3%)	166 (79.4%)
Total	96 (46.0%)	113 (54.0%)	209

**Legend**: M: malabsorption; I: intolerance; H_2_ positive: peak exceeded 20 parts per million (ppm) over the baseline; CH_4_ positive: peak > 10 ppm above baseline.

**Table 2:** Baseline and maximal values of H_2_ and CH_4_ in different groups.

	H_2_ +	H_2_ −	CH_4_ +	CH_4_ −
Baseline H_2_	21.3 (±22.1)	18.5 (±19.9)	15.1 (±12.5)	21.2 (±22.8)
Max H_2_ level	142.4 (±101)	21.2 (±21.4)	120.0 (±106.6)	59.3 (±80.8)
Delta H_2_	121 (±95.9)	2.7 (±14.7)	104.6 (±106.5)	38.1 (±72.9)
Baseline CH_4_	17.5 (±8.9)	18.8 (±12.7)	21.4 (±15.1)	17.2 (±9.3)
Max CH_4_	26.4 (±13.6)	22.0 (±15.9)	36.4 (±20.8)	19.8 (±9.5)
Delta CH_4_	8.9 (±8.9)	3.2 (±6.3)	15.0 (±10.7)	2.6 (±3.2)

**Legend:** max: maximal.

## Data Availability

Not applicable.
